# Expert consensus on vaccination in patients with inflammatory bowel disease in Japan

**DOI:** 10.1007/s00535-022-01953-w

**Published:** 2023-01-11

**Authors:** Takashi Ishige, Toshiaki Shimizu, Kenji Watanabe, Katsuhiro Arai, Koichi Kamei, Takahiro Kudo, Reiko Kunisaki, Daisuke Tokuhara, Makoto Naganuma, Tatsuki Mizuochi, Atsuko Murashima, Yuta Inoki, Naomi Iwata, Itaru Iwama, Sachi Koinuma, Hirotaka Shimizu, Keisuke Jimbo, Yugo Takaki, Shohei Takahashi, Yuki Cho, Ryusuke Nambu, Daisuke Nishida, Shin-ichiro Hagiwara, Norikatsu Hikita, Hiroki Fujikawa, Kenji Hosoi, Shuhei Hosomi, Yohei Mikami, Jun Miyoshi, Ryusuke Yagi, Yoko Yokoyama, Tadakazu Hisamatsu

**Affiliations:** 1 Department of Pediatrics, Gunma University Graduate School of Medicine, 3-39-22, Showa-Machi, Maebashi, Gunma 371-8511 Japan; 2 Department of Pediatrics and Adolescent Medicine, Juntendo University Graduate School of Medicine, Tokyo, Japan; 3 Division of Gastroenterology and Hepatology, Department of Internal Medicine, Hyogo Medical University, Nishinomiya, Japan; 4 Division of Gastroenterology, Center for Pediatric Inflammatory Bowel Disease, National Center for Child Health and Development, Tokyo, Japan; 5 Division of Nephrology and Rheumatology, National Center for Child Health and Development, Tokyo, Japan; 6 Department of Pediatrics, Juntendo University Faculty of Medicine, Tokyo, Japan; 7 Inflammatory Bowel Disease Center, Yokohama City University Medical Center, Yokohama, Japan; 8 Department of Pediatrics, Wakayama Medical University, Wakayama, Japan; 9 Department of Gastroenterology and Hepatology, Kansai Medical University, Osaka, Japan; 10 Department of Pediatrics and Child Health, Kurume University School of Medicine, Kurume, Fukuoka Japan; 11 Center for Maternal-Fetal, Neonatal and Reproductive Medicine, National Center of Child Health and Development, Tokyo, Japan; 12 Department of Infection and Immunology, Aichi Children’s Health and Medical Center, Obu, Japan; 13 Division of Gastroenterology and Hepatology, Saitama Children’s Medical Center, Saitama, Japan; 14 Japan Drug Information Institute in Pregnancy, National Center of Child Health and Development, Tokyo, Japan; 15 Department of Pediatrics, Japanese Red Cross Kumamoto Hospital, Kumamoto, Japan; 16 Department of Pediatrics, Kyorin University School of Medicine, Tokyo, Japan; 17 Department of Pediatrics, Osaka Metropolitan University Graduate School of Medicine, Osaka, Japan; 18 Department of Pediatric Gastroenterology, Nutrition and Endocrinology, Osaka Women’s and Children’s Hospital, Osaka, Japan; 19 Division of Gastroenterology, Tokyo Metro Children’s Medical Center, Tokyo, Japan; 20 Department of Gastroenterology, Osaka Metropolitan University Graduate School of Medicine, Osaka, Japan; 21 Division of Gastroenterology and Hepatology, Department of Internal Medicine, Keio University School of Medicine, Tokyo, Japan; 22 Department of Gastroenterology and Hepatology, Kyorin University School of Medicine, Tokyo, Japan; 23 Department of Intestinal Inflammation Research, Hyogo College of Medicine, Nishinomiya, Hyogo Japan

**Keywords:** Ulcerative colitis, Crohn’s disease, Immunization, Vaccine-preventable disease

## Abstract

Immunosuppressive therapies can affect the immune response to or safety of vaccination in patients with inflammatory bowel disease (IBD). The appropriateness of vaccination should be assessed prior to the initiation of IBD treatment because patients with IBD frequently undergo continuous treatment with immunosuppressive drugs. This consensus was developed to support the decision-making process regarding appropriate vaccination for pediatric and adult patients with IBD and physicians by providing critical information according to the published literature and expert consensus about vaccine-preventable diseases (VPDs) [excluding cervical cancer and coronavirus disease 2019 (COVID-19)] in Japan. This consensus includes 19 important clinical questions (CQs) on the following 4 topics: VPDs (6 CQs), live attenuated vaccines (2 CQs), inactivated vaccines (6 CQs), and vaccination for pregnancy, childbirth, and breastfeeding (5 CQs). These topics and CQs were selected under unified consensus by the members of a committee on intractable diseases with support by a Health and Labour Sciences Research Grant. Physicians should provide necessary information on VPDs to their patients with IBD and carefully manage these patients’ IBD if various risk factors for the development or worsening of VPDs are present. This consensus will facilitate informed and shared decision-making in daily IBD clinical practice.

## Introduction

Vaccination protects vaccinated persons and those around them who are vulnerable to disease, reducing the risk of disease spread among family members, colleagues, and other people in the community. The recent progress in the treatment of inflammatory bowel disease (IBD) has allowed more patients to receive immunosuppressive treatments (Table 1) than in the past. Because immunosuppressants often affect the safety and immunogenicity of vaccination and are often difficult to discontinue, the necessity of vaccination should be evaluated immediately after the diagnosis of IBD. This consensus was established to provide critical information required for clinicians to plan appropriate preventive care for patients with IBD. The information in the consensus is based on a literature review and the opinions of experts in IBD and vaccination.

**Table 1:** List of immunosuppressive and non-immunosuppressive treatments defined in this consensus

Immunosuppressive agents
Corticosteroids (prednisolone, methylprednisolone, budesonide)
Azathioprine
6-Mercaptopurine
Tacrolimus
Cyclosporine
Infliximab (including biosimilar)
Adalimumab (including biosimilar)
Golimumab
Ustekinumab
Tofacitinib
Non-immunosuppressive agents
5-ASA
Sulfasalazine
Enteral nutrition formula
Granulocyte and monocyte adsorption apheresis/ leukocytapheresis
Darvadstrocel
Vedolizumab^a^

*5-ASA* 5-aminosalicylic acid

^a^Vedolizumab is not included among the immunosuppressive agents because its effect is gut-selective, whereas specific consideration for opportunistic infections is recommended

Because of the lack of sufficient data, most of the previous guidelines on vaccination of patients with IBD considered only limited evidence of vaccine safety and effectiveness in IBD populations. In addition, evidence from individual countries is needed because routine vaccine schedules and sanitation measures vary from country to country; however, such evidence is scarce. Because close attention is essential to avoid vaccine-related adverse events [e.g., infection by the vaccine virus after administration of a live attenuated vaccine (LAV)], these consensus statements are based on reviews of manuscripts, accumulation of evidence, and discussion among members; however, they are not based on assessment of the certainty of the evidence. This consensus consists of the following sections: vaccine-preventable diseases (VPDs), LAVs, inactivated vaccines, and vaccination during pregnancy and breastfeeding. Information about severe acute respiratory syndrome coronavirus 2 (SARS-CoV-2) vaccination is provided elsewhere. Likewise, human papilloma virus vaccination is not described in this consensus because at the time of this writing, the recommendation for the vaccine had been temporarily suspended until October 2021.

Although this consensus is based on the infectious disease status, vaccination policy, and health insurance system in Japan, it will facilitate informed and shared decision-making in daily IBD clinical practice.

## Methods

A Health and Labour Sciences Research Grant for Research on Intractable Diseases from the Ministry of Health, Labour, and Welfare of Japan was obtained to support the development of a task force for establishment of an expert consensus on vaccination in patients with IBD. First, a steering committee comprising seven pediatric gastroenterologists, four physician gastroenterologists, and one expert in women’s health and medication during pregnancy was formed. The committee was charged to develop questions for a systematic literature search. The literature review team then performed the systematic literature search using PubMed and Ichushi (www.jamas.or.jp). Gray literature, including alerts from the government or statistical data from the National Institute of Infectious Diseases, was included. The review team comprised 20 physicians, including pediatric gastroenterologists, physician gastroenterologists, and women’s health experts. The review team was divided into four sections; vaccine-preventable diseases (VPDs), LAVs, inactivated vaccines, and vaccination during pregnancy and breastfeeding and made drafts of commentaries. The commentaries were discussed with the steering committee members, who were also divided into those sections, and statements were established. A Web meeting attended by all the committee members was then conducted to establish a consensus among the members. After a few corrections were made based on the discussion at the meeting, agreement among all the committee members was confirmed electronically. The manuscript was made available to all the intractable diseases grant members for commenting before submission. However, the strength of the recommendations was not adjudicated in this consensus.

## Vaccine-preventable diseases

**Fig. 1:**
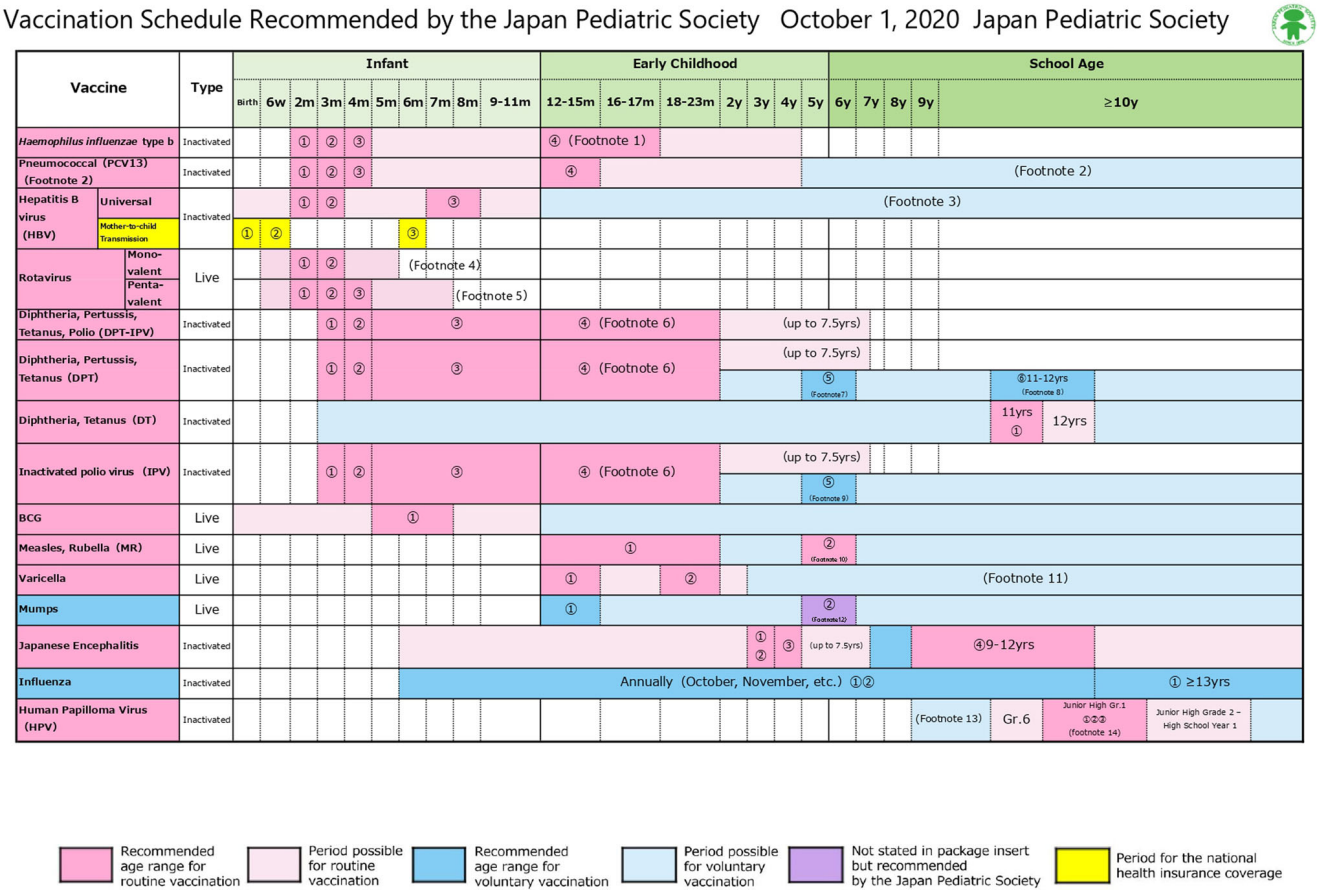
Standard vaccine schedule for children in Japan (as of October 2020). These recommendations are derived from the Japan Pediatric Society


VPDs are diseases that are preventable by vaccination. Table 2 and Fig. 1 show the currently available vaccines and the recommended schedule of vaccination in Japan. Infants and people of advanced age are susceptible to VPDs and tend to have more severe outcomes of VPDs compared with healthy adults. Patients with IBD also have an increased risk of the development or worsening of VPDs than do healthy individuals; therefore, physicians should provide necessary information on VPDs to their patients with IBD and carefully manage these patients if various risk factors for the development or worsening of VPDs are present (e.g., advanced age or current treatment with immunosuppressive therapies). Before deciding to vaccinate a patient with IBD, it is necessary to obtain the patient’s vaccination record and history of VPDs. Antibody testing should then be taken into consideration, especially for hepatitis B, measles, rubella, mumps, and varicella, although such testing is not covered by health insurance in Japan.

**Table 2:** Vaccines available in Japan

Routine vaccination
Live attenuated vaccine
Bacille Calmette-Guerin (BCG)
Measles (or measles-rubella combined)
Rubella
Varicella rotavirus
Inactivated vaccine
Diphtheria, pertussis, tetanus, and polio (DPT-IPV)
Diphtheria, pertussis, tetanus (DPT)
Polio
Diphtheria-tetanus (DT)
Japanese encephalitis
Human influenza type b (Hib)
Hepatitis B
Human papilloma virus (2- and 4-valent vaccines)
Influenza
Pneumococcus (13- and 23-valent vaccines)
COVID-19 (mRNA and viral vector vaccines)

Voluntary vaccination
Live attenuated vaccine
Mumps
Yellow fever
Shingles (live attenuated varicella)
Inactivated vaccine
Tetanus
Diphtheria
Hepatitis A
Meningococcus
Rabies
Shingles (recombinant zoster)
Human papilloma virus (9-valent vaccine)

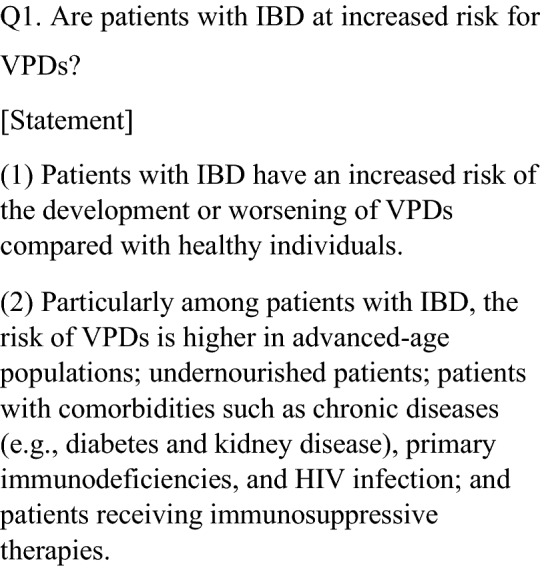


[Commentary]

Among patients with IBD, the risk of opportunistic infections is higher in advanced-age populations; undernourished patients; patients with comorbidities such as chronic diseases (e.g., diabetes and kidney disease), primary immunodeficiency, and HIV infection; and patients receiving immunosuppressive therapy [1–3]. In particular, immunosuppressive therapy increases the risk of opportunistic infections, and the immunosuppressive therapy-mediated risk of infection is known to be even higher in patients receiving combination therapies (e.g., thiopurines plus steroids or thiopurines plus steroids plus infliximab) [1–3]. In addition, individuals of advanced age sometimes do not know their vaccination record in spite of their increased risk of infection; thus, it is very important to prevent older patients with IBD from developing VPDs of an infectious nature.

In a systematic review, the pooled incidence of invasive pneumococcal disease was 65/100,000 person-years in patients with chronic inflammatory diseases (including IBD) compared with 10/100,000 in healthy controls [4]. In terms of *Haemophilus influenzae* type b (Hib), a cohort study showed no significant difference in the Hib-mediated mortality rate between IBD and non-IBD groups, but hospitalizations due to Hib infection were significantly higher in patients with than without IBD with an adjusted odds ratio of 1.34 [95% confidence interval (CI) 1.16–1.55] [5]. In terms of herpes zoster, which has been preventable by recombinant herpes zoster vaccine for populations of advanced age (≥ 50 years) since January 2020 in Japan, a cohort study demonstrated that the prevalence of herpes zoster in patients with IBD was significantly higher than that of healthy subjects [6, 7]. With respect to human papillomavirus disease, a systematic review of five cohort studies and three case–control studies clarified that patients with IBD on immunosuppressive therapy had an increased risk of cervical high-grade dysplasia/cancer compared with the general population [8]. In a comparative study, the long-term hospitalization rate due to seasonal influenza was significantly higher in patients with than without IBD (5.40% vs. 1.85%, respectively; *P* < 0.001) [9]. Several studies of hepatitis B virus (HBV) revealed a higher risk of HBV infection in patients with IBD than in healthy individuals [10, 11], whereas other studies showed no significant difference in HBV infection between individuals with and without IBD [12, 13]. The Japan Society of Hepatology guidelines for the management of HBV infection cautions clinicians about the risk of HBV reactivation in HBV carrier patients receiving immunosuppressive therapy; the guidelines thus recommend testing patients for HBV antibody prior to immunosuppressive therapy [14]. There is no evidence indicating a higher risk of diphtheria, tetanus, pertussis, or polio in patients with IBD than in healthy populations, possibly because of the low prevalence of these diseases or uneven distribution of cases [1].

No studies have analyzed the risks of measles, rubella, and mumps between patients with IBD and healthy individuals, but one study revealed a higher risk of severe measles infection in immunocompromised patients than in healthy individuals [15]. In terms of varicella zoster virus (VZV), one report revealed 5 deaths among 20 patients with IBD (16 adults and 4 children) receiving immunosuppressive therapy [16]. In a retrospective study of pediatric patients with IBD, the rate of hospitalization following a primary VZV infection was higher in patients with than without IBD [17]. A Japanese nationwide study that collected data on episodes of measles, rubella, varicella, and mumps in pediatric patients receiving immunosuppressive therapy for kidney disease, rheumatoid arthritis, gastrointestinal disease, or post-organ transplantation showed that 4 of 47 hospitalized pediatric patients (43 with VZV and 4 with mumps) receiving immunosuppressive therapy died of disseminated VZV infection [18]. This finding indicated a risk of worsening VZV infection in patients with IBD receiving immunosuppressive therapy. In terms of rotavirus, no study has analyzed the risks and severity of rotavirus infection between patients with IBD and healthy individuals. However, a case–control study of 4584 patients with rotavirus infection showed that immunocompromised patients had a 7.4-fold higher risk of hospitalization than the general population [19].
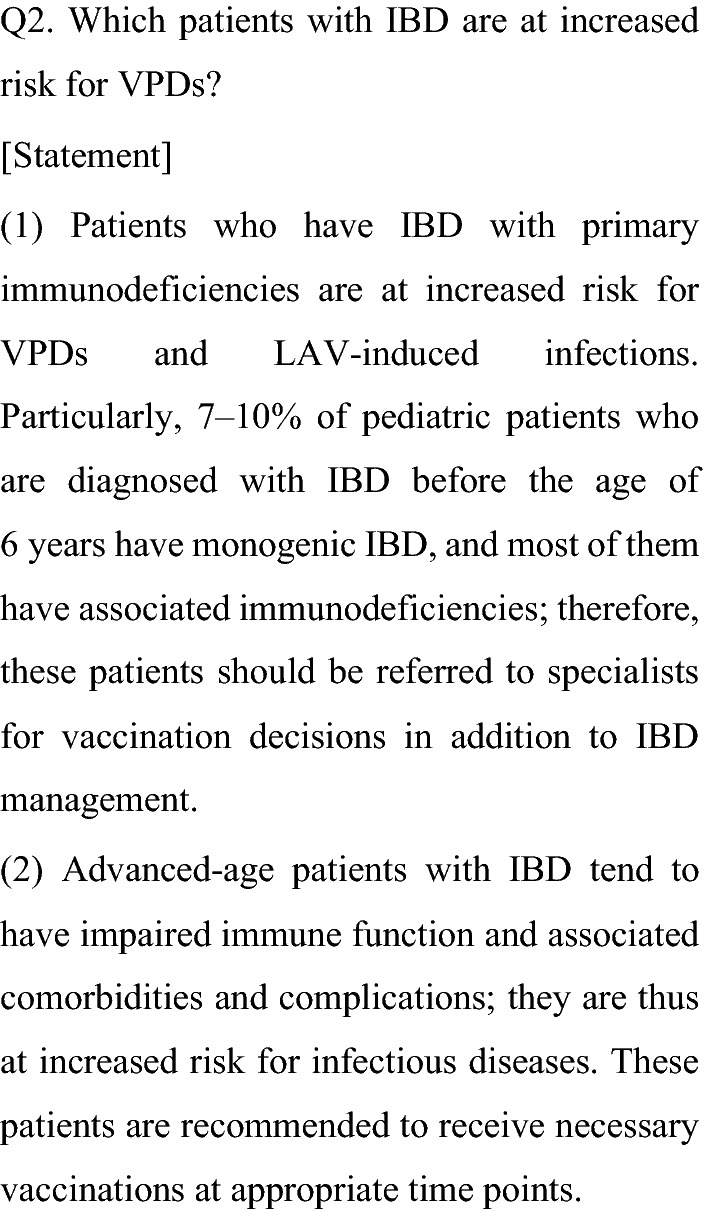


[Commentary]

Patients who have IBD with primary immunodeficiency and advanced-age patients with IBD are at high risk for VPDs. Primary immunodeficiency is a broad category of diseases in which immune resistance to pathogens is impaired at birth, and more than 400 different types of primary immunodeficiencies have been identified [20]. IBD caused by monogenic germline mutations (i.e., monogenic IBD) was recently found to be present in approximately 7–10% of patients with IBD diagnosed before the age of 6 years, and most of these cases of monogenic IBD were found to be comorbid with primary immunodeficiency disease [21]. In one systematic review, 44.7% of 750 patients with monogenic IBD had severe or atypical infections [22]. In Japan, targeted genetic panel tests for certain causative genes have been covered by national health insurance since 2018, making genetic diagnosis possible.

Vaccination programs for patients with IBD vary widely depending on the primary immunodeficiency disease. For example, LAVs are contraindicated in patients with primary immunodeficiency diseases affecting cellular immunity (such as severe combined immunodeficiency and Wiskott–Aldrich syndrome) because of the risk of life-threatening vaccine-induced illness. However, inactivated vaccines can be administered to patients with primary immunodeficiency, although the antibody levels after vaccination vary depending on the etiology of the primary immunodeficiency [23]. In patients with chronic granulomatous disease, Bacille Calmette-Guérin (BCG) is contraindicated because BCG vaccination may lead to the development of disseminated BCG disease, resulting in severe outcomes [24].

Therefore, if patients with IBD are suspected to have monogenic germline mutations or primary immunodeficiency because of very early onset (< 6 years of age), a family history of IBD or primary immunodeficiency, refractory anal lesions, or strong resistance to conventional IBD treatment, referral of these patients to specialized facilities for appropriate vaccination programs and schedules is recommended.

Advanced-age patients with IBD are at higher risk for VPDs than younger patients with IBD and healthy advanced-age individuals because of their reduced immunity and higher prevalence of comorbidities/complications such as diabetes, renal impairment, and low nutritional status [25]. Generally, individuals ≥ 65 years of age are at increased risk for invasive pneumococcal infection and seasonal influenza virus, and those ≥ 50 years of age are at increased risk for reactivation of VZV [26–28]. Immunosuppressive therapy has been shown to increase the risk of severe infections in individuals of advanced age. One study demonstrated that advanced-age patients with IBD receiving infliximab and adalimumab had a 20-fold higher incidence of severe infections than did patients of the same age with IBD but without infliximab and adalimumab therapy [29]. In terms of herpes zoster as an adverse event associated with tofacitinib therapy, tofacitinib-treated patients of advanced age (≥ 65 years) with ulcerative colitis have been shown to have higher rates of herpes zoster with an odds ratio of 9.55 (95% CI 4.77–17.08) than tofacitinib-treated younger patients (< 65 years of age) [30]. In particular, physicians should be aware of the risk of VPDs, including opportunistic infections, and should take into consideration appropriate vaccination planning in patients with refractory IBD receiving combinations of steroids, thiopurines, and biologic agents.
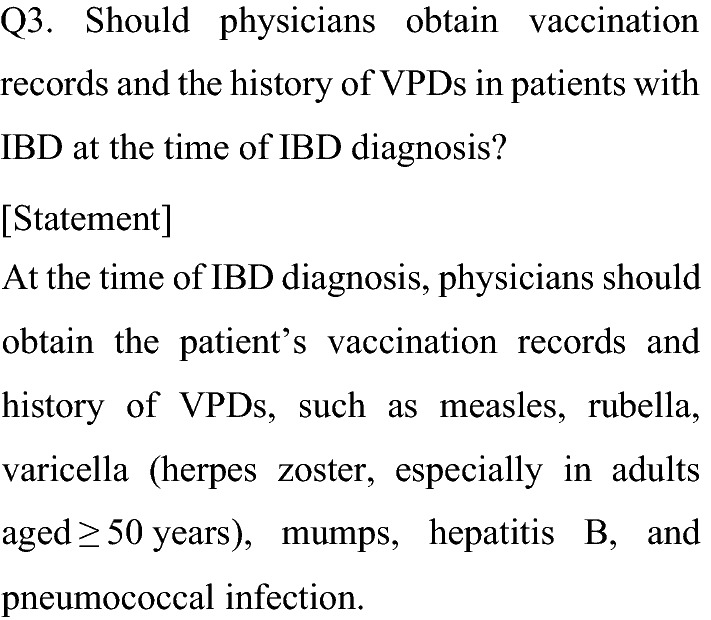


[Commentary]

Patients with IBD are at risk of developing opportunistic infections and aggravating VPDs during treatment depending on age, nutritional status, and immunosuppressive therapy [31–36]. Nevertheless, evaluation of immunity to VPDs seems to be insufficient in patients with IBD [37, 38].

To the extent possible, LAVs against VPDs (e.g., measles, rubella, varicella, and mumps) should be given before starting immunosuppressive therapy in patients with IBD [1]. For this purpose, it is necessary to confirm the vaccination records and history of VPDs at the time of IBD diagnosis. In particular, knowing the history of varicella or herpes zoster is important to determine the risk of developing herpes zoster while receiving immunosuppressive therapy. To prevent herpes zoster in patients of advanced age, recombinant herpes zoster vaccine has been available to adults aged ≥ 50 years since 2020 in Japan. Thus, it is also important to confirm the vaccination history of herpes zoster vaccine in advanced-age patients with IBD.

A program for HBV as a routine vaccination in infants has been in place since 2016 in Japan; thus, there remains a substantial HBV-unvaccinated population at risk of sexually transmitted HBV at or after adolescence. Patients receiving immunosuppressive therapy are at risk of HBV reactivation and aggravation of HBV infection [39, 40], and it is, therefore, necessary to obtain the HBV vaccination history and provide appropriate information to patients at the time of IBD diagnosis. In addition to HBV, it is appropriate to obtain all of the patient’s vaccination history to fully understand the risk of aggravation of other VPDs.
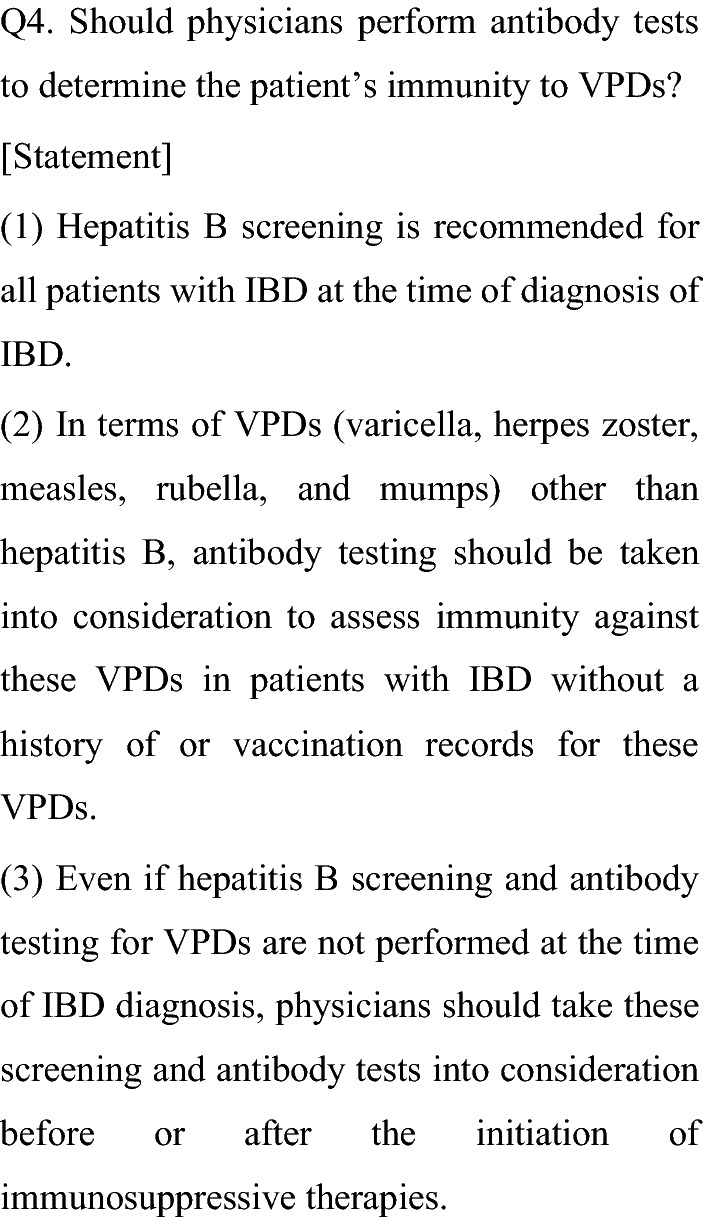


[Commentary]

Several cohort studies worldwide have indicated that the prevalence of HBV and hepatitis C virus (HCV) in patients with IBD is similar to that in the general population [2, 41, 42]. Reactivation of HBV is a well-known complication of immunosuppression, and an increased incidence of liver failure due to viral reactivation has been described in patients with IBD under immunosuppression [39, 40]. The European Crohn’s and Colitis Organisation (ECCO) guideline recommends serological HBV screening for all patients at diagnosis of IBD [2], and Pittet et al. [43] recommended serological HBV screening including hepatitis B surface (HBs) antigen, anti-HBs immunoglobulin G (IgG), and anti-hepatitis B core IgG for adults with IBD in Switzerland. Although HCV is not a VPD, the guidelines recommend serological screening for HCV using antibody testing in patients with IBD at diagnosis. If possible, HCV-RNA testing should be taken into consideration for HCV screening [2, 44].

Patients with IBD who are undergoing immunosuppressive therapy and seronegative for VZV IgG are at risk of severe varicella and thus require prompt post-exposure prophylaxis in the event of exposure. At diagnosis of IBD, patients should be screened for their susceptibility to primary varicella infection by obtaining a thorough history. We suggest anti-herpes zoster IgG antibody testing for patients without a clear history of varicella, herpes zoster, or receipt of varicella vaccine [2]. We also suggest testing for antibodies to measles, rubella, mumps, and VZV at diagnosis of IBD at least once, although as of 2021, insurance coverage of antibody testing is not approved for patients other than those who are suspected to have infection in Japan [43].

At diagnosis of IBD, or at least prior to commencement of immunosuppressive therapy including vedolizumab, we suggest testing for antibodies to HBV, VZV, measles, rubella, and mumps. For seronegative patients with IBD, we suggest administration of the course of vaccines.
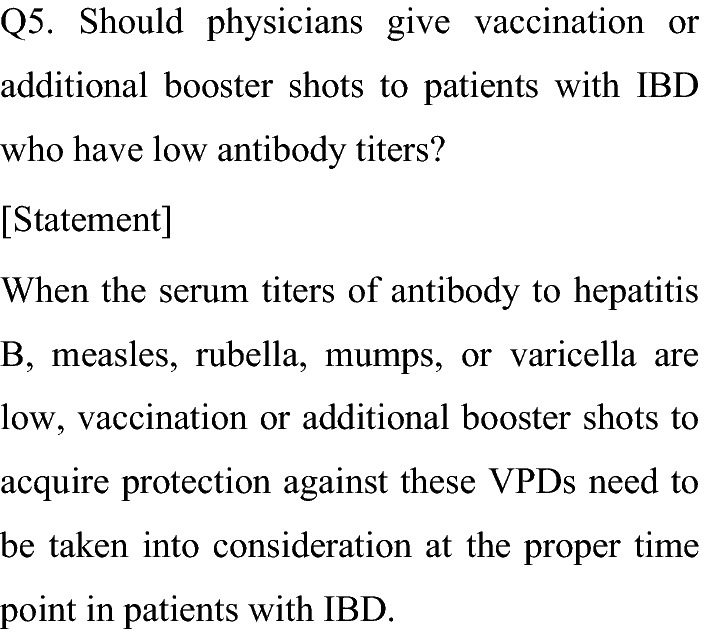


[Commentary]

Patients with IBD have increased risks of opportunistic infections and development or worsening of VPDs according to their age (e.g., advanced age), nutritional status, and immunosuppressive therapies. Therefore, maintaining protective immunity in patients with IBD by vaccination is critical to optimize these patients’ outcomes [1, 2, 25, 38, 43, 45–49]. For patients with IBD not on immunosuppressive therapy, we suggest administration of live and inactivated vaccines. For patients on immunosuppressive therapy, LAVs are not recommended [1, 45]. In terms of HBV vaccine for patients with IBD, we suggest administration of a three-dose vaccination series (at 0, 1, and 6 months) when the anti-HBs antibody titer declines to < 10 mIU/mL. When the anti-HBs antibody titer is still < 10 mIU/mL at 1–2 months after a three-dose vaccination series in patients with IBD, we suggest administration of an additional three-dose vaccination series. For patients with an immune response to HBV vaccine (anti-HBs antibody titer of ≥ 10 mIU/mL), we suggest no further antibody testing and no booster shots [14, 50]. The test sensitivity and the cut-offs for protective immunity to measles, rubella, and VZV vary among the techniques used, such as enzyme immunoassays [enzyme immunoassay (EIA) and enzyme-linked immunosorbent assay (ELISA)], hemagglutination inhibition assays, particle agglutination assays, and neutralization tests [50]. Based on an understanding of these problems, we suggest using the antibody titers necessary to consider the need for vaccinations that are documented in several domestic guidelines for vaccination of patients with IBD. For example, based on a guideline for vaccination from the Japan Society for Hematopoietic Cell Transplantation, the ELISA IgG antibody titers necessary to consider the need for vaccinations against measles, rubella, and varicella are ≤ 4.0, ≤ 5.0, and ≤ 5.0 IU/mL, respectively [51]. In another study and guideline, the EIA IgG antibody titer necessary to consider the need for vaccinations against measles, rubella, mumps, and varicella was ≤ 4.0 for all [52]. The United States Centers for Disease Control and Prevention (CDC) stated that the sensitivity of ELISA to detect antibody to VZV varies according to the antigens used and that ELISA is, therefore, not accurate enough for evaluation of immunity against VZV. The CDC recommends purified glycoprotein ELISA as a more sensitive method; however, testing with purified glycoprotein ELISA is not commercially available. Thus, it is necessary to appropriately interpret the examined VZV antibody titers based on an understanding of these problems raised by commercial assays.

Although the sensitivities and cut-off points of antibody titers vary according to the testing methods, we suggest administration of vaccination or booster shots to patients with IBD when the ELISA (or EIA) IgG antibody titers are < 4–5 against measles, rubella, mumps, and varicella. In terms of the Hib vaccine, herpes zoster vaccine (recombinant vaccine), influenza vaccine, *Streptococcus pneumoniae* vaccine, meningococcal vaccine, human papillomavirus vaccine, and diphtheria, tetanus, and pertussis vaccine combined with inactivated poliovirus (DTaP-IPV) vaccine, we suggest that vaccination is based not on the antibody titers but instead on the previous vaccination records [45, 49, 51].
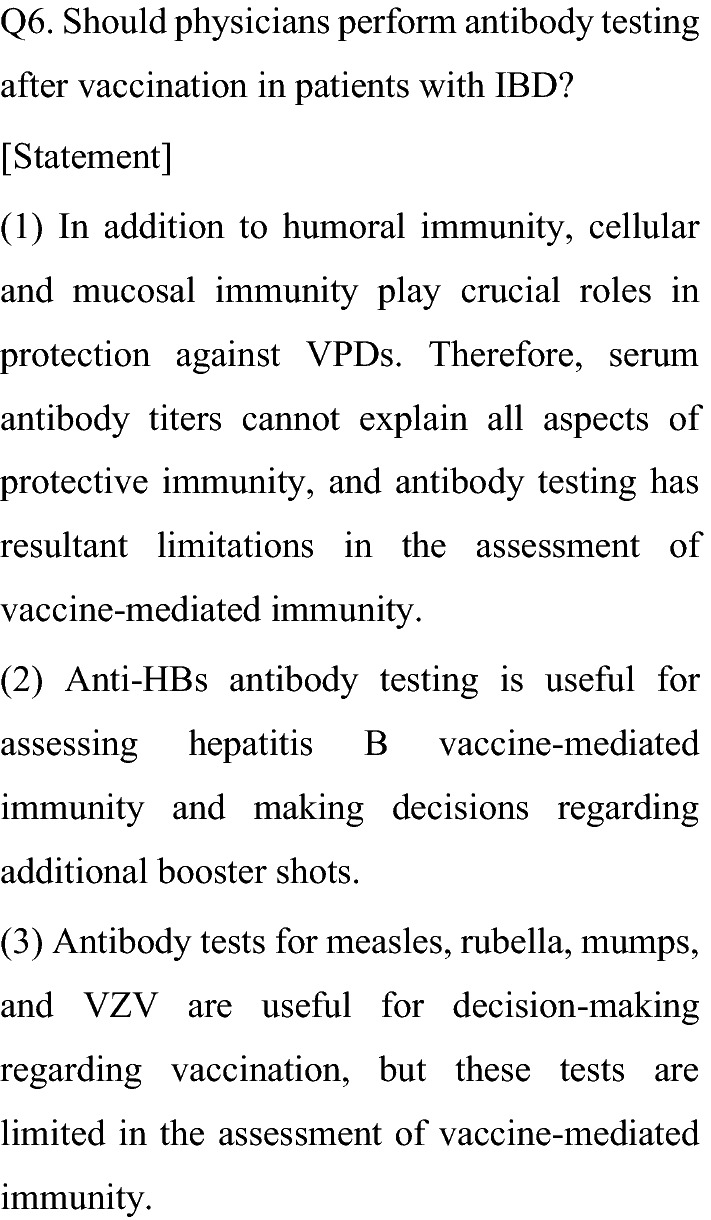


[Commentary]

Not only humoral immunity with antibodies but also physical barriers (e.g., mucus and epithelial cells) at the site of infection of pathogenic microorganisms and various immune cells involved in mucosal immunity are important in the prevention of infection. In other words, it is difficult to predict the severity of the infection or whether it can be prevented based only on antibody titers [53], therefore; evaluation of the immune status after vaccination should be judged comprehensively based on each patient’s age, nutritional status, and administration of drugs such as immunosuppressive agents. Measuring antibody titers after vaccination or during the course of IBD treatment is relatively easy and objective and can be used to make decisions on additional vaccination. Notably, however, the sensitivity of antibody measurement and the optimal cutoff value of the antibody for prevention of infection vary depending on the measurement technique.

Regarding HBV, the Canadian Association of Gastroenterology and the ECCO recommend that all patients with IBD receive a three-dose series of HBV vaccinations and that the anti-HBs IgG antibody titer be measured 4–12 weeks after the third vaccination to achieve an anti-HBs antibody level of > 10 IU/L [2, 45]. HBV vaccination of all infants has been routine in Japan since 2016 to prevent horizontal transmission, but people born before that date (excluding those born to HBs antigen-positive mothers) are unlikely to have HBV immunity. It is thus important to confirm patients’ birth year and vaccination records. The vaccination guideline for healthcare workers issued by the Japanese Society for Infection Prevention and Control also recommends confirmation of the anti-HBs IgG antibody titer after a three-dose series of HBV vaccination and, if necessary, consideration of an additional booster to achieve an anti-HBs antibody level of ≥ 10 IU/L [50].

Regarding measles, rubella, and mumps, the CDC does not recommend serologic testing pre- and post-vaccination because of the low sensitivity of such testing to detect antibodies [54]. There is reportedly no difference in antibody levels after vaccination for measles, rubella, and mumps viruses between patients with IBD on immunosuppressive therapy and healthy controls [55]. Consequently, measuring antibody titers after vaccination in these patients might be useful to determine primary vaccine failure, in which a person does not develop protective immunity after vaccination, or secondary vaccine failure, in which a person loses an initially acquired immunity [56]. However, the usefulness of routine antibody titer measurement is limited because the rate of antibody acquisition after vaccination is generally very high.

With regards to VZV, the CDC does not recommend pre- and post-vaccination serologic testing because of the low sensitivity of antibody detection after vaccination [57]. Because no correlations between the viral antibody titer and the risk of developing herpes zoster or the severity of herpes zoster/postherpetic neuralgia have been reported [58, 59], the usefulness of antibody titer measurement after vaccination is low.

## Live attenuated vaccines

LAVs are made of pathogenic viruses or bacteria with reduced toxicity. Because they contain pathogenic organisms, the risk of infection due to the vaccine strain cannot be eliminated. As a rule, therefore, immunocompromised or immunosuppressed patients and pregnant woman should not receive LAVs because of safety concerns.

In this section, immunosuppressive treatment that requires attention with respect to the concomitant use of LAVs will be explored. Furthermore, the recommended duration between the last LAV and introduction of immunosuppressive treatment as well as the duration between the cessation of immunosuppressive treatment and LAV administration will be discussed.

LAVs for pregnant women will be discussed in another section. Yellow fever vaccine, which is administered to individuals who travel to endemic areas, will not be discussed; its risks and benefits should be considered in the clinical setting as needed.

Although live varicella vaccine is approved for the prevention of herpes zoster in people aged ≥ 50 years, the recently approved inactivated herpes zoster vaccine can be used when an LAV is contraindicated.

Vaccinating family members and surrounding individuals could decrease the risk of infection in patients who should not receive LAVs.
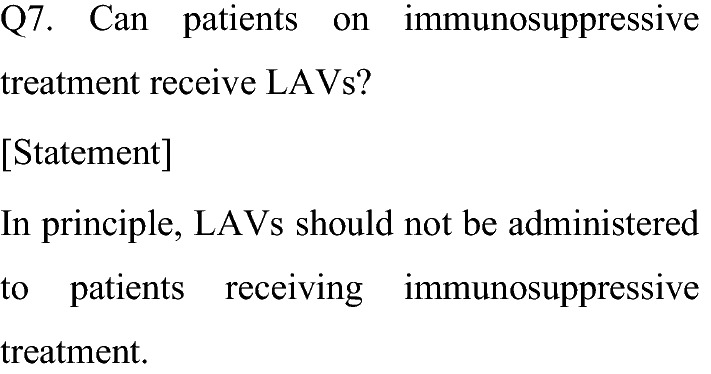


[Commentary]

Effectiveness of LAVs in patients receiving immunosuppressive treatment

Clinical data regarding the efficacy of LAVs in patients receiving immunosuppressive treatment are limited [1, 2]. However, several prospective interventional studies have focused on the resultant antibody elevation after LAV administration in patients who have undergone organ transplantation and in those receiving immunosuppressive treatments, including biologics.

In a Japanese study, the seroconversion rate for measles, rubella, varicella, and mumps vaccines was 80.0%, 100%, 59.1%, and 62.9%, respectively, in children and young adults receiving immunosuppressants for IBD or other conditions if their immunological condition met the criteria of a CD4 cell count of ≥ 500/mm^3^, stimulation index of phytohemagglutinin-induced lymphocyte proliferation of ≥ 101.6, and serum IgG level of ≥ 300 mg/dL. Among 32 patients, only one 5-year-old boy with Crohn’s disease receiving azathioprine developed a breakthrough varicella infection [52]. Another study revealed the probable efficacy of live varicella vaccines for adults with IBD receiving azathioprine [60].

A large prospective randomized controlled study (VERVE trial) of the efficacy of live varicella vaccine in patients receiving anti-tumor necrosis factor (anti-TNF) agents is ongoing in the United States.

Accumulation of further evidence for the efficacy of LAVs in patients receiving immunosuppressive treatment is expected.

Safety of LAVs in patients receiving immunosuppressive treatment

Administration of LAVs to patients with IBD receiving immunosuppressive treatment in Japan should follow the package insert of each drug (Table 3) as well as the “Immunization guideline for children post-organ transplantation and under immunosuppressive treatment 2014” [61]. Thus, in principle, LAVs are not recommended for patients receiving immunosuppressive treatments other than vedolizumab. However, clinical research on the use of LAVs in patients receiving immunosuppressive treatment has been conducted in several countries, including Japan, and a consensus and social system to support the use of LAVs in patients who may benefit from them are needed.

**Table 3:** Description of vaccination in the package insert for the treatment of inflammatory bowel disease

Trade name	Generic name	Prescribing information
Steroids		
PREDONINE	Prednisolone Prednisolone Sodium succinate	Important precautions: do not administer live vaccines to patients receiving long-term or high-dose steroids or within 6 months after discontinuation
PREDONEMA Enema	Prednisolone Sodium phosphate	
STERONEMA	Betamethasone Sodium phosphate	
RINDERON suppositories	Betamethasone	
Solu-medrol for intravenous use	Methylprednisolone Sodium succinate	Contraindications: do not administer live vaccines or attenuated live vaccines to patients receiving immunosuppressive therapy of this medicine
RECTABUL rectal foam	Budesonide	Important precautions: when administering a live vaccine to a patient receiving this medicine, the patient's immunological condition should be examined, and extreme caution should be exercised
Zentacoat	Budesonide	None stated
Immunomodulators		
IMURAN/AZANIN	Azathioprine	Contraindications for co-administration: there is a risk of breakthrough infection in immunosuppressed patients who receive live vaccines. The live vaccine may increase and become pathogenic if administered to
LEUKERIN	Mercaptopurine Hydrate	
Immunosuppressants		
Prograf	Tacrolimus Hydrate	Contraindications for co-administration: do not inoculate live vaccines
Sandimmun	Ciclosporin	
METHOTREXATE	Methotrexate	Important precautions: do not inoculate live vaccines while receiving this medicine
Biological products		
REMICADE	Infliximab	Important precautions: do not inoculate live vaccines while receiving this medicine Pregnant women: caution should be exercised when live vaccines are administered to infants born to patients who have received this medicine
HUMIRA	Adalimumab	Important precautions: do not inoculate live vaccines Pregnant women: caution should be exercised when live vaccines are administered to infants born to patients who have received this medicine
Simponi	Golimumab	Important precautions: do not inoculate live vaccines while receiving this medicine Pregnant women: caution should be exercised when live vaccines are administered to infants born to patients who have received this medicine
Stelara	Ustekinumab	Important precautions: do not inoculate live vaccines while receiving this medicine
ENTYVIO	Vedolizumab	Precautions for co-administration: if symptoms based on the live vaccine appear after vaccination, appropriate measures should be taken. The possibility of infection caused by live vaccines cannot be ruled out
XELJANZ	Tofacitinib citrate	Important precautions: do not inoculate live vaccines while receiving this medicine

In principle, LAVs are not recommended for patients with immunosuppressive conditions, particularly cellular immune deficiency, because of the risk of lethal viral infection by the vaccine strain. Therefore, LAVs are contraindicated in patients undergoing immunosuppressive treatment. Some lethal events after administration of LAVs, including yellow fever and BCG vaccines, have been reported in this population [62, 63].

Although a few reports have addressed the safety of LAVs for patients with IBD receiving immunosuppressants, the safety of measles, rubella, varicella, and mumps was discussed in a large systematic review and other reports [52, 60, 64–66]. The BCG and rotavirus vaccines are supposed to be given before 1 year of age. Patients with infantile-onset IBD receiving immunosuppressive treatment and infants born of mothers receiving immunosuppressive treatment require special attention. Primary immunodeficiency should be ruled out in patients with infantile-onset IBD, and in principle, LAVs should not be administered in this high-risk population [61]. The “Pregnancy and delivery” section addresses the care of infants born of mothers receiving immunosuppressive treatment. No reports have described the administration of BCG and rotavirus vaccine to infants with IBD receiving immunosuppressive treatment.

Patients with IBD are thought to be at high risk of VPDs [67, 68]. Patients with very-early-onset IBD under immunosuppressive treatment are at particularly high risk if they miss or receive an insufficient number of immunizations. They may not receive contraindicated vaccines, such as LAVs.

In general, patients receiving immunosuppressive therapy are at high risk of serious infection [31], and prevention of VPDs should be emphasized; however, LAVs cannot be administered if the instructions in the package inserts are followed. To change this situation, an ongoing study is evaluating the safety and efficacy of LAVs in patients under immunosuppressive treatment [52]. Although the acceptable immunological condition for safe LAV administration in patients under immunosuppressive treatment requires further evaluation, studies have suggested that LAVs can be safely administered if these patients’ cellular and humoral immunological parameters are within normal levels.
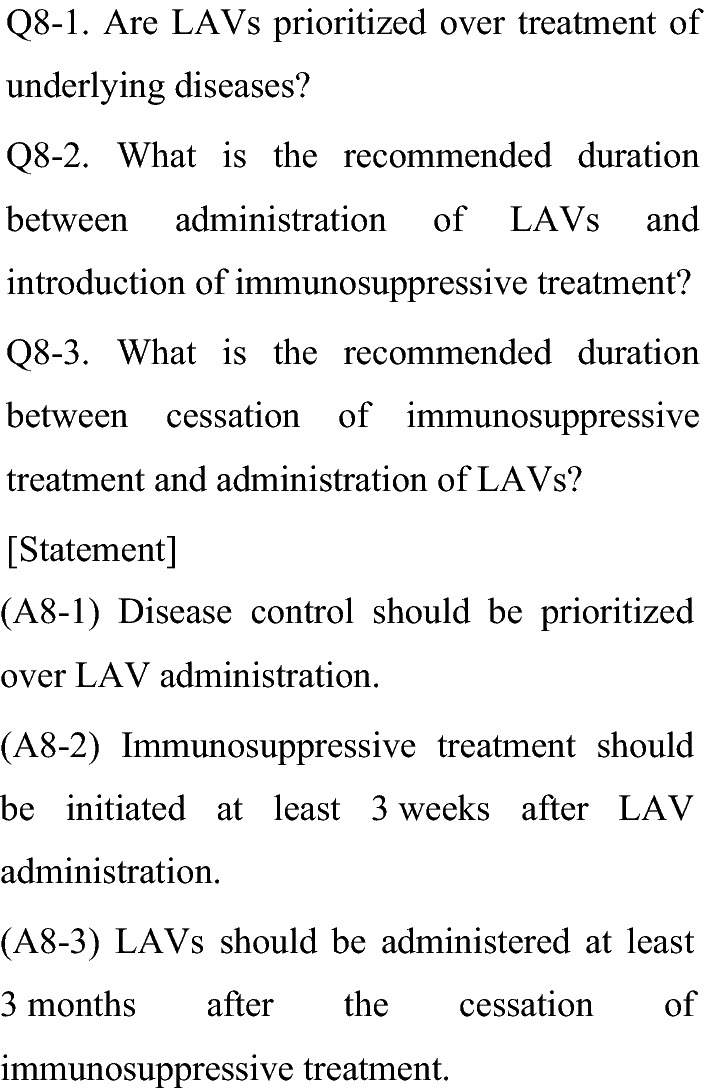


[Commentary]

LAVs for patients without prior administration and without detectable antibody should be discussed on an individual-patient basis considering the disease activity and importance of vaccination. Ideally, LAVs should be administered before the introduction of immunosuppressive treatment [2, 61]. However, some patients with IBD require immunosuppressive treatment before completion of LAVs. Disease control should be prioritized in those requiring early immunosuppressive treatment, and administration of LAVs should be considered once immunosuppressive treatments are discontinued in the disease course [1, 69]. LAVs should be administered once disease remission has been induced without immunosuppressants.

**Fig. 2:**
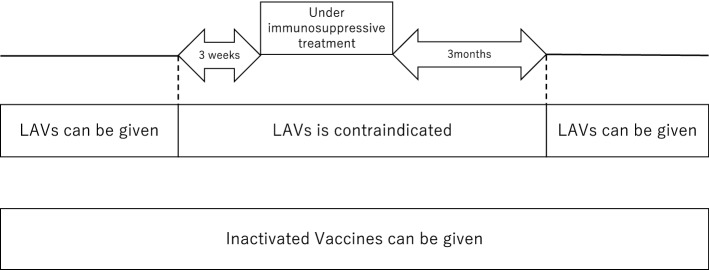
Duration between immunosuppressive treatment and live attenuated vaccine administration [61]

Immunosuppressants should be initiated at least 1 month after LAV administration according to the ECCO guideline and at least 3 weeks after LAV administration according to the “Immunization guideline for children undergoing organ transplantation and immunocompromised condition” (Fig. 2) [2].

For patients under immunosuppressive treatment, LAVs can be administered at least 3 months after immunosuppressive treatment is discontinued as written in the domestic guideline (Fig. 2) [61]. Those taking high-dose corticosteroids should not receive LAVs for 6 months after the discontinuation of steroids according to the package inserts. The ECCO guideline recommends a 1-month duration between the cessation of immunosuppressive treatments (corticosteroids, methotrexate, tofacitinib, cyclosporine, and tacrolimus) and LAV administration based on the half-life of these immunosuppressants. The package insert of each LAV does not clearly indicate the duration between cessation of immunosuppressants and LAV administration.

Although infection by the vaccine virus of an LAV could occur in patients under immunosuppressive treatment, there are few reports of lethal or serious infection except for patients receiving BCG [63]. Therefore, the risks and benefits of LAV administration in high-risk patients should be considered on an individual-patient basis.

## Inactivated vaccines

Inactivated vaccines are made by isolating and purifying disease-causing pathogens, such as bacteria and viruses, and inactivating them with formalin, phenol, heat treatment, or ultraviolet irradiation to eliminate their infectivity and pathogenicity without affecting their protective antigens. Therefore, serious adverse reactions are unlikely to occur. However, compared with LAVs, immunity is more difficult to acquire and is shorter-lasting. Therefore, repeated vaccinations are required to maintain immunity, followed by additional immunizations after a certain period of time. A toxoid is a vaccine in which bacterial toxins are treated with formalin or other agents to eliminate toxicity. These vaccines require repeated inoculations similar to inactivated vaccines. Table 2 shows a list of inactivated vaccines and toxoids that can be provided in Japan.

In daily medical practice, inactivated vaccines and toxoids are often collectively referred to as inactivated vaccines; thus, the term “inactivated vaccines” is used in this section to refer to both types.

Inactivated vaccines for patients with IBD are considered effective except in cases of severe disease or use of high-dose steroids. Furthermore, inactivated vaccines can be administered during immunosuppressive therapy. Notably, the possibility of a low antibody acquisition rate should be considered.

This section describes the relevance of inactivated vaccines to IBD.
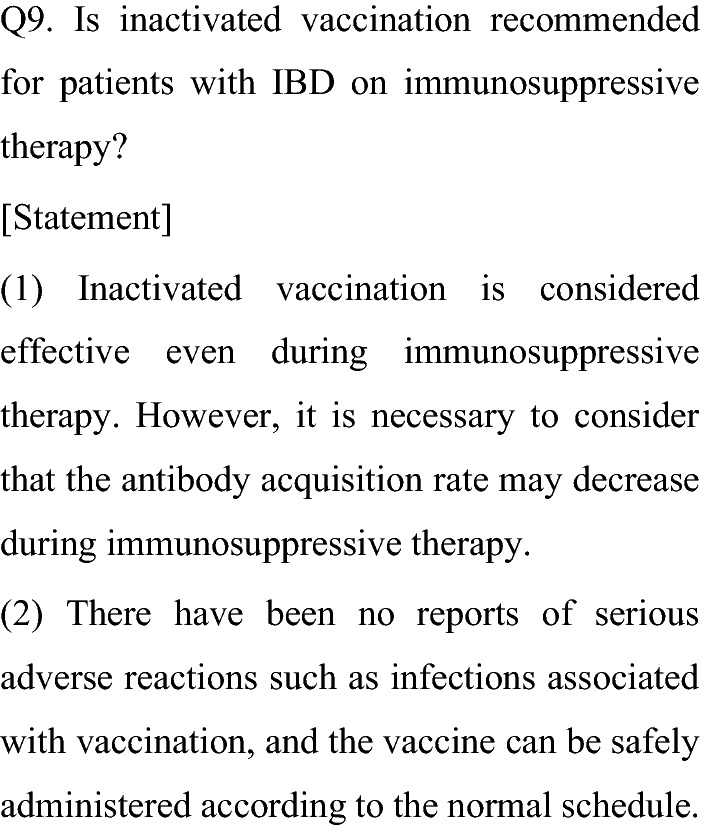


[Commentary]

Several reports have described inactivated vaccination of patients with IBD on immunosuppressive therapy, including reviews [70, 71]. Large numbers of patients have acquired antibodies, and the safety of this practice has been deemed adequate.

Influenza virus vaccines can reportedly reduce the rate of immune acquisition in patients treated with thiopurines or anti-TNF agents [72, 73].

In patients receiving the hepatitis B vaccine, immunomodulatory drugs such as azathioprine and methotrexate did not decrease the antibody acquisition rate; the acquisition rate reportedly decreased only in patients with IBD using anti-TNF agents [74]. In addition, one study showed that older age is associated with a lower antibody acquisition rate [75].

Patients with IBD have a high incidence of cervical and oral cancer, and patients with anal lesions are at high risk of human papillomavirus-related anal cancer. Therefore, antibody acquisition by human papillomavirus vaccination is important. In a report of the human papillomavirus tetravalent vaccine in 33 pediatric patients with IBD on immunosuppressive therapy, 100% of the patients acquired antibodies to types 6, 11, and 16 and 96% acquired antibodies to type 18 [76]. These rates were comparable to the antibody acquisition rate in healthy women, and there were no serious adverse reactions related to vaccination [76].

In one study, the median seroprotection rate against the pneumococcal 13-valent diphtheria conjugate vaccine (PCV13) significantly increased from 43.9% at inclusion to 90.4% (*P* < 0.001) after vaccination. Patients receiving anti-TNF agents achieved a slightly lower seroprotection rate (from 44.5 to 86.6%) than patients treated with other types of immunosuppressive therapy [77]. Furthermore, patients administered infliximab or combination immunosuppressive therapy had significantly lower response rates against the 23-valent pneumococcal polysaccharide vaccine (PPSV23) (57.6% and 62.5%, respectively) compared with the group on mesalamine (88.6%; *P* < 0.05 for both comparisons) [78]. Additionally, both PCV13 and PSSV23 were generally safe and well tolerated.

Regarding the safety of the vaccine, a systematic review of pediatric patients with IBD revealed no infections caused by the vaccine strain, indicating that the vaccine can be safely administered [71].

Inactivated vaccines can be safely administered to patients with IBD on immunosuppressive therapy according to the normal schedule, with most patients acquiring antibody.
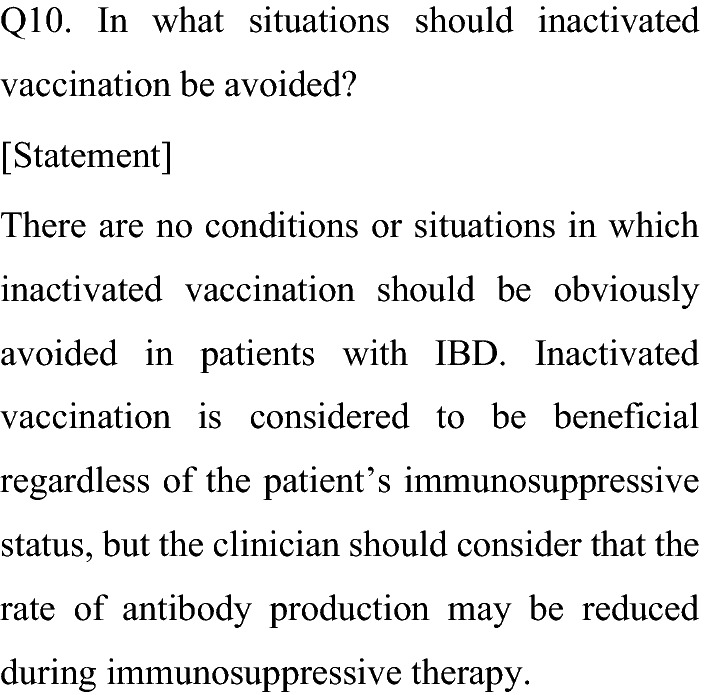


[Commentary]

Inactivated vaccines can be administered to patients with IBD who are not receiving immunosuppressive therapy, with the same safety considerations as for healthy patients. However, a systematic review of pediatric patients with IBD showed that even those on immunosuppressive therapy can be safely vaccinated without developing infections caused by vaccine strains [71]. The same results were reported in a systematic review of pediatric rheumatic disorders, not IBD [79]. Immunosuppressive therapy causes a decrease in both cellular (T-cell) and humoral (B-cell) immunity. In the latter case, both live and inactivated vaccines are available. In the former case, LAVs are contraindicated in principle, and inactivated vaccines may cause a reduction in antibody production because the helper T cells may not be able to reproduce the antibody-producing function of B cells.

A decrease in the immune response due to immunosuppressive therapy can reportedly occur when prednisolone is administered at a dose of ≥ 2 mg/kg/day (≥ 20 mg/day for 10 kg) for longer than 14 days at a body weight of < 10 kg or when thiopurines or biologic agents are used [80]. However, the same judgment cannot be made to every patients because the mechanism of action and concomitant conditions of the drugs used, immunological characteristics of individual patients, nutritional status, and other factors also affect the results.

Several reports have described a reduction in inactivated vaccine-acquired antibody titers during immunosuppressive therapy. In a study of influenza virus vaccines, the seroconversion rate was lower in pediatric patients with IBD treated with thiopurines and infliximab than in healthy subjects [81].

Conversely, a retrospective cohort study of the vaccine in pediatric patients and a randomized controlled study in adult patients showed that a sufficient antibody production rate was obtained even during immunosuppressive therapy in pediatric patients [82], whereas a sufficient antibody production rate was observed in adult patients other than those using anti-TNF agents [73]. In addition, research has suggested that the efficacy of HBV and pneumococcal vaccines may be attenuated during the acute phase of the disease and during immunosuppressive therapy [83].
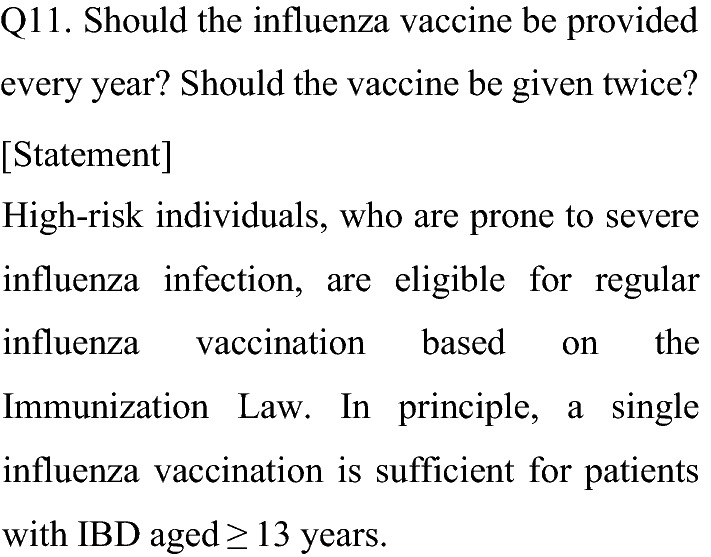


[Commentary]

Influenza is an acute febrile infectious disease caused by the influenza virus. The symptoms are often associated with high fever, regardless of the vaccination status; the severity of the disease is often mild to moderate; and the prognosis is generally relatively good. The disease often resolves spontaneously with a fatality rate of < 0.1%. Although the effectiveness of the vaccine varies among seasons, vaccination is recommended by the Japanese Association for Infectious Diseases for patients at high risk of complications from influenza (Table 4). In patients with IBD receiving immunosuppressive therapy, active vaccination is recommended because such patients are considered to be at high risk of complications.

**Table 4:** Patients at high risk of complications during influenza infection

6-Month-old or more and under 5-year-old
65 Years of age or older
Chronic respiratory diseases (bronchial asthma, COPD, etc.)
Cardiovascular disease (excluding hypertension alone)
Chronic kidney, liver, blood, metabolic diseases (diabetes, etc.)
Neuromuscular disease (including motor paralysis, convulsions, dysphagia)
Immunosuppressive state (including those caused by HIV and drugs)
Pregnancy
Residents of long-term medical treatment facilities
Significant obesity
Patients receiving long-term aspirin
Cancer-bearing patients

*COPD* chronic obstructive pulmonary disease, *HIV* human immunodeficiency virus

Guidelines from the Japanese Association for Infectious Diseases and from the United States recommend that influenza vaccination be administered by the end of October [84]. The prevalent influenza strains vary each year. In addition, the vaccine’s effectiveness in prevention of the disease is strongest after vaccination and decreases by approximately 8–9% each subsequent month. Therefore, annual influenza vaccination is recommended.

In individuals aged ≥ 65 years, the benefit of the vaccine is decreased at 30 days post-vaccination. One study showed that the rate of reduced efficacy in patients of this age was 10.8% (95% CI 2.6–23.8%) for influenza A (H3N2), 9.6% (95% CI − 3.3 to 32.7%) for influenza A (H1N1), and 10.8% (95% CI 1.4–33.9%) for influenza B/Yamagata. Notably, antibody titers are more likely to decrease in patients of advanced age because of the slightly faster rate of reduction than in the overall population of patients aged ≥ 18 years [84]. Not only patients of advanced age but also pregnant women, children under 5 years of age, and patients with underlying medical conditions should be cautious because of the possibility of influenza complications [85].

A randomized comparative study of the influenza vaccine in adults with IBD undergoing anti-TNF therapy or thiopurine therapy was conducted by dividing the patients into two groups: those who received a single dose and those who received an additional dose. There was no difference in the immune response after additional vaccination with the influenza vaccine, and no additional vaccination was required [86].

The Japanese Association for Infectious Diseases recommends a single dose of influenza vaccine for people aged ≥ 13 years and two doses for children aged ≤ 12 years.
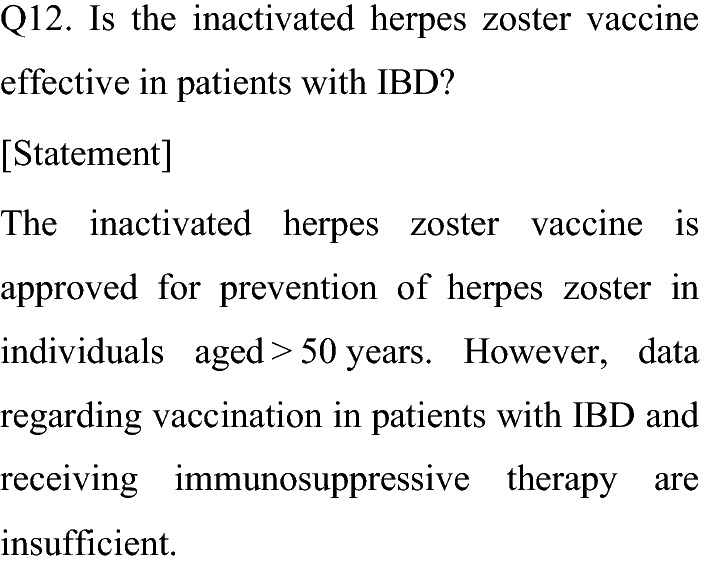


[Commentary]

Herpes zoster is a skin disease caused by VZV reactivation. It is characterized by erythema and neuralgia that usually develop eccentrically. Postherpetic neuralgia is characterized by hyperalgesia and allodynia that persist even after herpes zoster has resolved, and many cases of intractable chronic pain have been recorded. Consequently, patients’ quality of life markedly decreases.

The only way to prevent herpes zoster and postherpetic neuralgia is inoculation with the vaccine [87–89]. In Japan, in addition to the dry attenuated live varicella vaccine, recombinant herpes zoster vaccine (an inactivated vaccine) can be used in adults aged > 50 years. This inactivated vaccine has a preventive effect of ≥ 90% against herpes zoster and postherpetic neuralgia with two doses at an interval of 2–6 months, and > 85% of cases can be prevented for 4 years after vaccination [90, 91]. Therefore, the inactivated herpes zoster vaccine is recommended to prevent complications associated with herpes zoster in immunized adults aged > 50 years regardless of their history of live vaccination or herpes zoster occurrence. This inactivated vaccine is also preferable to the LAVs for the prevention of herpes zoster and related complications.

Notably, the concomitant use of LAVs with steroids and thiopurines is contraindicated, and LAVs are not recommended for patients treated with biologics that affect the immune system. Instead, the inactivated herpes zoster vaccine is prescribed for these patients. The inactivated vaccine has been approved for adults aged > 50 years, but its efficacy is not fully confirmed in patients with IBD, especially those receiving immunosuppressive therapy [92]. Because inoculation with the inactivated herpes zoster vaccine is paid for by the patients themselves, it will be economically burdensome.
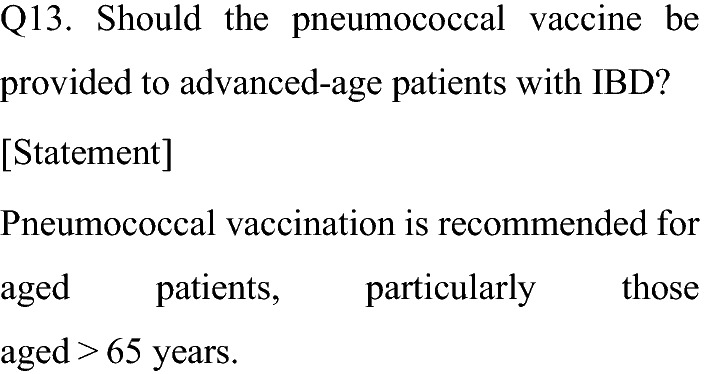


[Commentary]

In 2019 in Japan, the “Concept of *Streptococcus pneumoniae* vaccine for adults aged 65 and over” (3rd edition) was presented by the joint committee of the Japanese Respiratory Society and the Japanese Society of Infectious Diseases. In March 2021, the “Concept of pneumococcal vaccination for high-risk persons aged 6 to 64” was presented by the joint committee of the Japanese Respiratory Society, the Japanese Society of Infectious Diseases, and the Japanese Society of Vaccination.

Pneumococcal vaccines include PPSV23 and PCV13. PPSV23 is a routine vaccine for adults aged ≥ 65 years. Currently, PCV13 inoculation followed by a series of PPSV23 inoculation (regular or voluntary inoculation) can be administered. In Japan, the effect of the vaccine within 5 years after PPSV23 inoculation is 27.4% for all types of pneumococcal pneumonia and 33.5% for serum-type pneumococcal pneumonia [93]. According to a report targeting invasive pneumococcal disease in patients aged ≥ 15 years in Japan, the effect of vaccination against invasive pneumococcal disease caused by the serotypes included in the PPSV23 vaccine is 45%. Age-stratified analysis revealed that the efficacy of the PPSV23 vaccine serotype is 75% in individuals aged 15–64 years and 39% in individuals aged > 65 years. Based on this evidence, an improvement in the PPSV23 inoculation rate in adults aged ≥ 65 years has been expected; nevertheless, vaccination for individuals with underlying diseases has not been investigated. Additionally, pneumococcal vaccines for high-risk persons (such as patients with rheumatoid arthritis and collagenous diseases) aged 6–64 years have been presented by the joint committee, indicating the need to recognize attenuation of immunogenicity of the pneumococcal vaccine in patients undergoing thiopurine therapy.

Although clear evidence on whether IBD increases the risk of pneumococcal infection has not been provided [45], the pneumococcal vaccine is recommended for adult patients with IBD who are considered to be at a high risk of pneumococcal infection or who are receiving immunosuppressive therapy. This vaccine should also be administered before immunosuppressants are given. However, studies have yet to verify whether pneumococcal vaccination is recommended for patients with IBD if immunosuppressive treatment is not performed or for patients with fewer risk factors for pneumococcal infection. Data are also insufficient to propose the most appropriate type of vaccine and administration schedule.

Age is considered a risk factor for pneumococcal infection; thus, pneumococcal vaccination is beneficial for older patients with IBD, especially those aged ≥ 65 years, unless they have contraindications for vaccination.
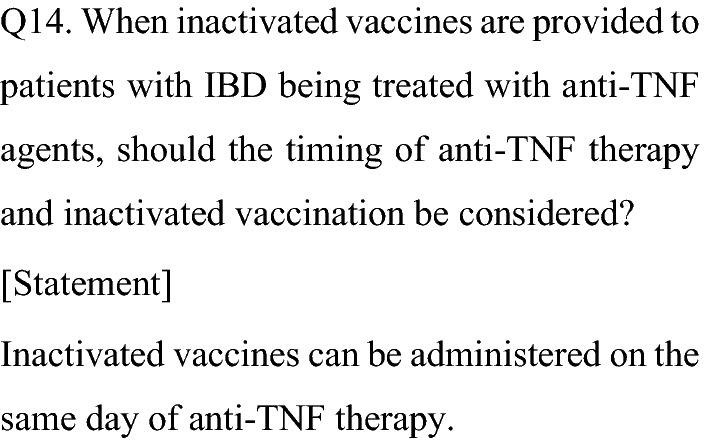


[Commentary]

Several studies have revealed the efficacy of inactivated vaccination for patients who have IBD, rheumatoid arthritis, and other collagenous diseases receiving anti-TNF agents. Although the antibody acquisition rate of the vaccine is slightly reduced in patients using anti-TNF agents [81, 94], sufficient antibody titers have been obtained, and inoculation can be safely conducted. These findings show that the benefits of inactivated vaccination outweigh its disadvantages. Therefore, inactivated vaccines can be administered to patients with IBD even when they are using anti-TNF agents.

Few studies have described the appropriate timing of inactivated vaccination after anti-TNF agents are given. In a study of patients with rheumatoid arthritis and ankylosing spondylitis being treated with infliximab, the antibody acquisition rates were compared between patients inoculated with influenza vaccine on the day of infliximab administration (*n* = 22) and patients inoculated 3 weeks after infliximab administration (*n* = 16). The results indicated no difference in the antibody acquisition rates between the two groups [95]. Interestingly, in patients with rheumatoid arthritis, a higher immune response to vaccination against the H1N1 strain was achieved in the group inoculated on the day of infliximab administration than in the group inoculated 3 weeks after infliximab administration.

A randomized controlled trial of patients with IBD was conducted to compare patients inoculated on the day of infliximab administration (*n* = 69) with those inoculated between two doses of infliximab administration (*n* = 68). All patients received influenza vaccines [A/California/7/2009 (H1N1), A/Victoria/361/2011 (H3N2), B/Wisconsin/1/2010] while undergoing infliximab maintenance therapy. The proportions of patients with sufficient antibody titers to prevent influenza infection (hemagglutination inhibition antibody titer of ≥ 1:40) were 67% versus 66% (*P* = 0.8) for H1N1, 43% versus 49% (*P* = 0.5) for H3N2, and 69% versus 79% (*P* = 0.2) for B/Wisconsin/1/2010, respectively. In addition, no significant differences in the exacerbation of the current disease or in the incidence of adverse reactions were observed between the two groups, and no serious adverse events were observed in either group [82].

Research on influenza vaccines for patients taking infliximab is limited. However, some studies have indicated that there is no evidence showing that the schedule of inactivated vaccination should be staggered in patients receiving anti-TNF agents. Therefore, inactivated vaccines can be administered on the same day as anti-TNF agents.

## Pregnancy, lactation, and vaccination

Special consideration is required for pregnant and lactating patients with IBD. Vaccination and infection control are key issues during these periods. Because VPDs often affect pregnancy outcomes, vaccination should be appropriately received before pregnancy if possible. If a patient diagnosed with IBD wishes to become pregnant, vaccination should be administered at the time of IBD diagnosis.

The benefits and risks of vaccination during pregnancy must be carefully explained to patients. Inactivated vaccines are considered safe during pregnancy. For patients who are worried about vaccines, accurate information including the incidence of congenital malformations or spontaneous abortion among typical births should be provided. The indication for vaccines for infants born to mothers with IBD should be considered based on the duration, types, and placental transfer of immunosuppressive agents used during pregnancy.
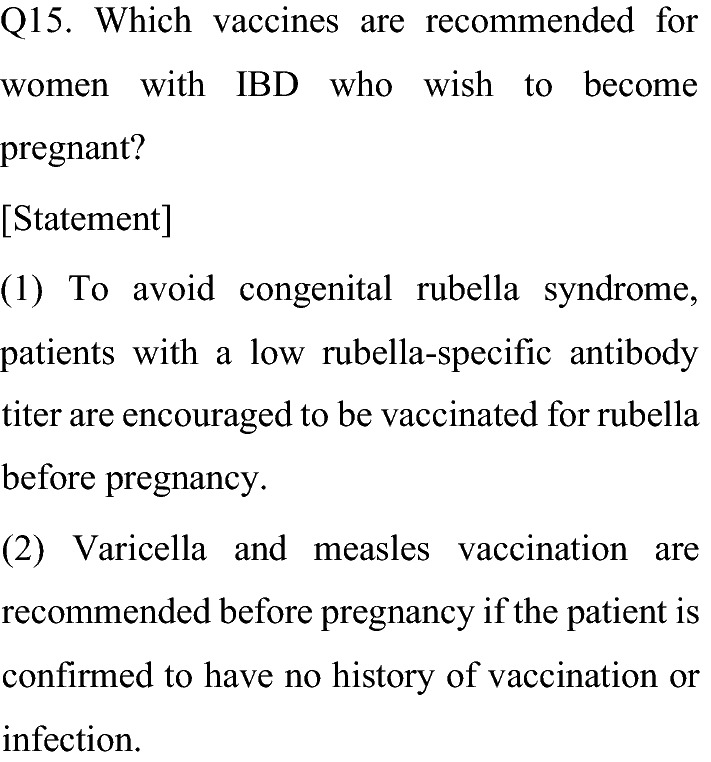


[Commentary]

A rubella epidemic occurred in Japan from 2012 to 2013, resulting in a high incidence of congenital rubella syndrome. The cause of this epidemic is considered to be the high rate of lack of sensitization to rubella among men in their 30 s and 40 s and young pregnant women at that time. A rubella epidemic and an outbreak of congenital rubella syndrome were also reported from 2018 to 2019 [96]. Infection with rubella in the first trimester of pregnancy can cause congenital rubella syndrome, potentially resulting in cataracts, glaucoma, congenital heart disease, and sensorineural hearing loss. Congenital rubella syndrome due to reinfection also may occur even if the mother has been previously vaccinated, although this is rare. In Japan, rubella vaccination is recommended to prevent the development of congenital rubella syndrome for women who wish to become pregnant if their rubella-specific antibody titer (hemagglutination inhibition method) is ≤ 1:16.

Because of the risk of severe disease if varicella or measles is contracted during pregnancy, women with IBD who have no history or vaccination of these diseases and wish to become pregnant should be considered for these vaccines. Because rubella vaccine, measles vaccine, measles-rubella vaccine, and varicella vaccine are LAVs, other sections of this document should be consulted to determine the vaccination plan for patients who are receiving and/or planning scheduled immunosuppressive treatment.

Because these vaccines are contraindicated during pregnancy, patients should be instructed to use contraception for 2 months after vaccination. If rubella or measles vaccines are administered to a patient who is unaware that she is pregnant or when pregnancy occurs within 2 months of vaccination, the CDC and the Japan Society of Obstetrics and Gynecology guidelines indicate that there is no need to interrupt the pregnancy because no clinically significant risk to the fetus has been demonstrated in previous research [97].

Voluntary rubella vaccination is recommended for partners of pregnant women, their children, and their other family members living together who have no history of rubella or vaccination. To prevent rubella transmission to the female partner, rubella vaccination is recommended for men with IBD who have no history or vaccination of rubella.
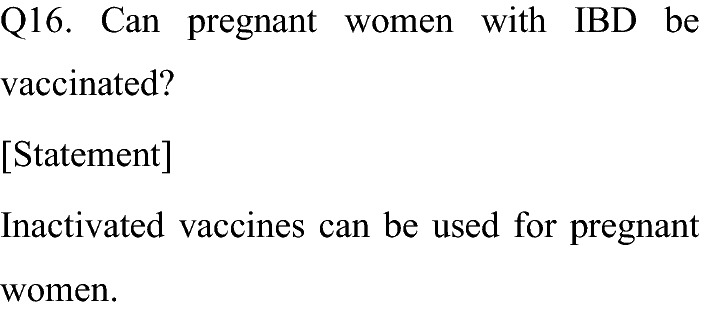


[Commentary]

In principle, inoculation of pregnant women using LAVs should be avoided because of concerns about the possibility of transfer of the vaccine component virus to the fetus, resulting in infection (see Q15). Vaccines other than LAVs (e.g., inactivated vaccines) do not cause infection in the fetus and can be administered as necessary.

Patients with IBD are considered at risk for severe influenza infection, and annual influenza vaccination is recommended in both Europe and the United States [2, 80]. Increased risks of the following have been reported in pregnant women who contract influenza: hospitalization, maternal mortality, spontaneous abortion, preterm birth, low birth weight, small for gestational age, and fetal death [98–101]. The influenza vaccine used in Japan is an inactivated vaccine, which theoretically poses no risk to pregnant women or fetuses. Many studies have revealed the safety of influenza vaccination during pregnancy, showing that it is not associated with adverse birth events and can improve pregnancy outcomes [102, 103]. Influenza vaccination of pregnant and postpartum women reduces the incidence of influenza in infants up to 6 months of age. In the United States, inactivated influenza vaccination of pregnant women is recommended during influenza epidemics because it is the most effective technique for preventing severe cases of influenza [104].

Based on the above, it is recommended that pregnant women with IBD, who are considered to be more susceptible to severe influenza, receive inactivated influenza vaccine when they desire vaccination.
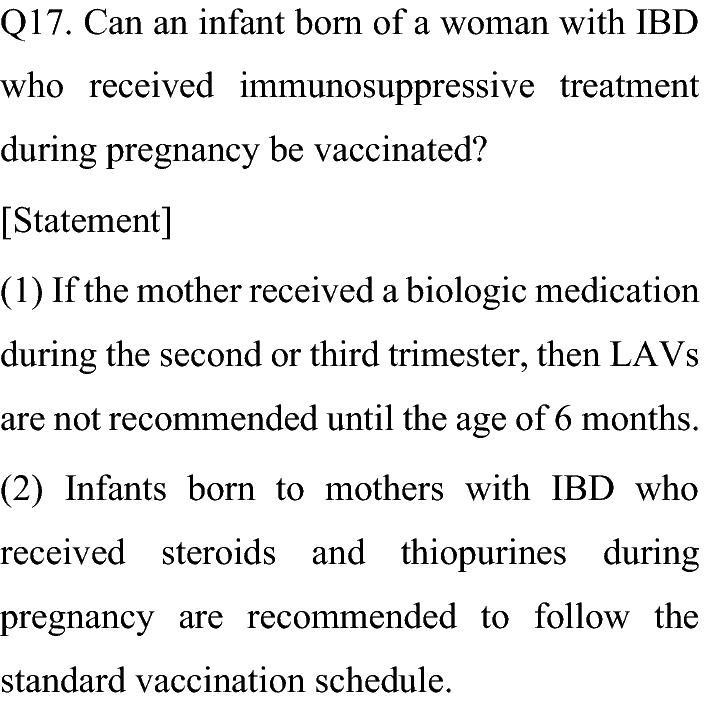


[Commentary]

Currently, four types of inactivated vaccines (Hib, pneumococcal vaccine, hepatitis B vaccine, and DTaP-IPV vaccine) and two LAVs (BCG and rotavirus) are recommended for standard vaccination at < 1 year of age in Japan (Fig. 1).

For many biologics, drug concentrations in cord blood are reportedly higher than those in maternal plasma (infliximab, about 1.6 times; adalimumab, about 1.5 times; vedolizumab, about 0.8 times; and ustekinumab, about 1.8 times) [105–107]. Because of the long plasma disappearance half-life of biologics, these drugs were still detectable in the blood at 3–6 months of age in infants born to mothers with IBD who had received infliximab and adalimumab in the third trimester of pregnancy [105, 108].

In a single case report describing a child of a mother exposed to infliximab during pregnancy, the child received a BCG vaccination at 3 months of age and then died of disseminated BCG infection at 4.5 months of age [63]. Although some reports have indicated that no major problems were observed in the children [109, 110], national and international professional guidelines recommend that infants of mothers who received biologics during the second or third trimester of pregnancy should avoid LAVs and BCG vaccination for at least 6 months after birth [61, 111–113].

Administration of the rotavirus vaccine is recommended by 14 weeks 6 days after birth to avoid vaccination at 6 months to 2 years of age, which is a susceptible period for the development of vaccination-associated intussusception. Therefore, it is difficult to actively recommend vaccination of infants born to mothers with IBD who received immunosuppressive treatment during pregnancy. Some reports have indicated no apparent increase in adverse reactions after rotavirus vaccination by 6 months of age in infants born to mothers with IBD who had received biologic agents (infliximab, adalimumab, ustekinumab, or vedolizumab) during pregnancy [114, 115].

Administration of inactivated vaccines should follow a standard vaccination schedule. Several reports have described an adequate immune response with no adverse events after inactivated vaccines were given to infants born to mothers with IBD who had received immunosuppressive treatment during pregnancy [110, 114, 116].

Placental transfer has been measured for prednisolone [117], thiopurines [118, 119], and immunosuppressive drugs (tacrolimus and cyclosporine) [120, 121], and the cord blood concentrations of all were reportedly lower than those in maternal plasma. Because the plasma elimination half-life of these drugs is shorter than that of biologics, the use of any of these agents until delivery is not considered to adversely affect either live or inactivated vaccines given according to the standard vaccination schedule in Japan [122].

One report described B-cell hypofunction in the cord blood of an infant born to a mother who had continued azathioprine during pregnancy, but the infant’s immune function was normal at 1 month of age [123]. Other reports have described blood cell abnormalities and decreased immune function in infants born to mothers who had continued azathioprine during pregnancy, and all of these abnormalities normalized within about 2 months [124, 125].
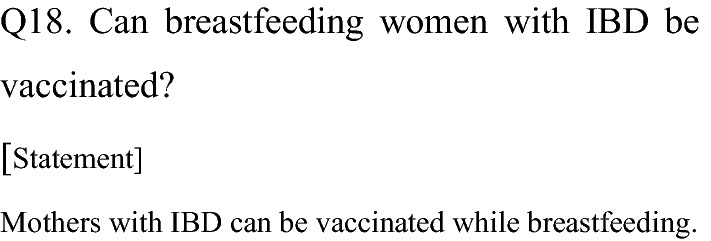


[Commentary]

In Japan, vaccination is considered to have no adverse effect on the safety of breast milk when live or inactivated vaccines are administered to nursing women [126]. Although components of the rubella vaccine are reportedly secreted into breast milk and transient asymptomatic infection has been observed in infants [127], most infants did not show clinical symptoms, and the rubella vaccination of the children was not affected [128, 129].

The yellow fever vaccine is rarely administered in Japan; however, some reports have described yellow fever vaccine-associated encephalitis and neurological disorders in infants after the vaccine was administered to lactating mothers [130, 131]. The CDC states that lactating mothers should be vaccinated only if travel to a yellow fever-endemic area is unavoidable [126].
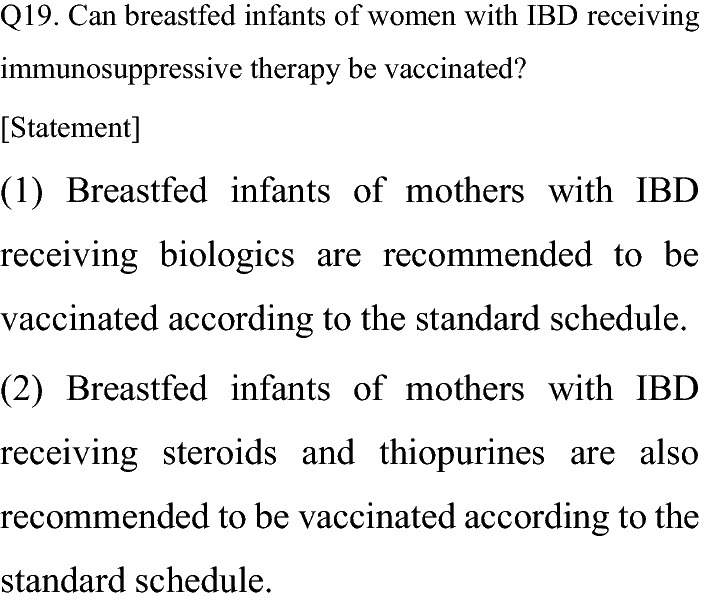


[Commentary]

Factors that enhance drug transfer to breast milk include low blood protein binding, basic drugs, high lipophilicity, and small molecular weight.

Biologics have very high molecular weights, which limits their transfer to breast milk. Infliximab and adalimumab have been detected in very small amounts by sensitive assays, but no reports have described adverse events in breasted infants [132–134]. Because the oral bioavailability of biologics is extremely low, even if a small amount of a drug is ingested orally by an infant through breast milk, it is hardly absorbed and thus poses no problem for the infant’s vaccination.

Prednisolone and thiopurines have been measured in the milk of breastfeeding women who are orally receiving these drugs, and the amount of drug ingested by fully lactating infants from breast milk is estimated to be about 1–5% of the infant’s therapeutic dose; additionally, the estimated rate of adverse events in lactating infants is low [135, 136]. Expert guidelines in North America and Europe consider prednisolone and thiopurines to be safe for use during breastfeeding [111, 112, 137–140]. Because of the low likelihood of affecting the infant’s immune function, general vaccination of infants is not considered problematic.

During steroid pulse therapy, the amount of drug ingested by the infant via breast milk increases with the dose of medication; therefore, the clinician must carefully consider whether breastfeeding should be performed. If a breastfed infant shows symptoms that suggest an adverse event caused by a drug transferred to breast milk, the infant’s drug blood level should be measured and immune function evaluated before vaccination.
