# Correction to: Expert consensus on vaccination in patients with inflammatory bowel disease in Japan

**DOI:** 10.1007/s00535-023-01965-0

**Published:** 2023-02-10

**Authors:** Takashi Ishige, Toshiaki Shimizu, Kenji Watanabe, Katsuhiro Arai, Koichi Kamei, Takahiro Kudo, Reiko Kunisaki, Daisuke Tokuhara, Makoto Naganuma, Tatsuki Mizuochi, Atsuko Murashima, Yuta Inoki, Naomi Iwata, Itaru Iwama, Sachi Koinuma, Hirotaka Shimizu, Keisuke Jimbo, Yugo Takaki, Shohei Takahashi, Yuki Cho, Ryusuke Nambu, Daisuke Nishida, Shin-ichiro Hagiwara, Norikatsu Hikita, Hiroki Fujikawa, Kenji Hosoi, Shuhei Hosomi, Yohei Mikami, Jun Miyoshi, Ryusuke Yagi, Yoko Yokoyama, Tadakazu Hisamatsu

**Affiliations:** 1 Department of Pediatrics, Gunma University Graduate School of Medicine, 3-39-22, Showa-Machi, Maebashi, Gunma 371-8511 Japan; 2 Department of Pediatrics and Adolescent Medicine, Juntendo University Graduate School of Medicine, Tokyo, Japan; 3 Division of Gastroenterology and Hepatology, Department of Internal Medicine, Hyogo Medical University, Nishinomiya, Japan; 4 Division of Gastroenterology, Center for Pediatric Inflammatory Bowel Disease, National Center for Child Health and Development, Tokyo, Japan; 5 Division of Nephrology and Rheumatology, National Center for Child Health and Development, Tokyo, Japan; 6 Department of Pediatrics, Juntendo University Faculty of Medicine, Tokyo, Japan; 7 Inflammatory Bowel Disease Center, Yokohama City University Medical Center, Yokohama, Japan; 8 Department of Pediatrics, Wakayama Medical University, Wakayama, Japan; 9 Department of Gastroenterology and Hepatology, Kansai Medical University, Osaka, Japan; 10 Department of Pediatrics and Child Health, Kurume University School of Medicine, Kurume, Fukuoka Japan; 11 Center for Maternal-Fetal, Neonatal and Reproductive Medicine, National Center of Child Health and Development, Tokyo, Japan; 12 Department of Infection and Immunology, Aichi Children’s Health and Medical Center, Obu, Japan; 13 Division of Gastroenterology and Hepatology, Saitama Children’s Medical Center, Saitama, Japan; 14 Japan Drug Information Institute in Pregnancy, National Center of Child Health and Development, Tokyo, Japan; 15 Department of Pediatrics, Japanese Red Cross Kumamoto Hospital, Kumamoto, Japan; 16 Department of Pediatrics, Kyorin University School of Medicine, Tokyo, Japan; 17 Department of Pediatrics, Osaka Metropolitan University Graduate School of Medicine, Osaka, Japan; 18 Department of Pediatric Gastroenterology, Nutrition and Endocrinology, Osaka Women’s and Children’s Hospital, Osaka, Japan; 19 Division of Gastroenterology, Tokyo Metro Children’s Medical Center, Tokyo, Japan; 20 Department of Gastroenterology, Osaka Metropolitan University Graduate School of Medicine, Osaka, Japan; 21 Division of Gastroenterology and Hepatology, Department of Internal Medicine, Keio University School of Medicine, Tokyo, Japan; 22 Department of Gastroenterology and Hepatology, Kyorin University School of Medicine, Tokyo, Japan; 23 Department of Intestinal Inflammation Research, Hyogo College of Medicine, Nishinomiya, Hyogo Japan

**Correction to: Journal of Gastroenterology (2023) 58:135–157** 10.1007/s00535-022-01953-w

The article “Expert consensus on vaccination in patients with inflammatory bowel disease in Japan”, written by Takashi Ishige, Toshiaki Shimizu, Kenji Watanabe, Katsuhiro Arai, Koichi Kamei, Takahiro Kudo, Reiko Kunisaki, Daisuke Tokuhara, Makoto Naganuma, Tatsuki Mizuochi, Atsuko Murashima, Yuta Inoki, Naomi Iwata, Itaru Iwama, Sachi Koinuma, Hirotaka Shimizu, Keisuke Jimbo, Yugo Takaki, Shohei Takahashi, Yuki Cho, Ryusuke Nambu, Daisuke Nishida, Shin-ichiro Hagiwara, Norikatsu Hikita, Hiroki Fujikawa, Kenji Hosoi, Shuhei Hosomi, Yohei Mikami, Jun Miyoshi, Ryusuke Yagi, Yoko Yokoyama, Tadakazu Hisamatsu, was originally published electronically on the publisher’s internet portal on 11 January 2023 without open access. With the author(s)’ decision to opt for Open Choice the copyright of the article changed on 17 January 2023 to © The Author(s) 2023 and the article is forthwith distributed under a Creative Commons Attribution 4.0 International License, which permits use, sharing, adaptation, distribution and reproduction in any medium or format, as long as you give appropriate credit to the original author(s) and the source, provide a link to the Creative Commons licence, and indicate if changes were made. The images or other third party material in this article are included in the article's Creative Commons licence, unless indicated otherwise in a credit line to the material. If material is not included in the article's Creative Commons licence and your intended use is not permitted by statutory regulation or exceeds the permitted use, you will need to obtain permission directly from the copyright holder. To view a copy of this licence, visit http://creativecommons.org/licenses/by/4.0/

